# Anti-Myxovirus Resistance Protein-1 Immunoglobulin A Autoantibody in Idiopathic Pulmonary Fibrosis

**DOI:** 10.1155/2022/1107673

**Published:** 2022-03-29

**Authors:** Toru Arai, Masaki Hirose, Yoshimasa Hamano, Tomoko Kagawa, Akihiro Murakami, Hiroshi Kida, Atsushi Kumanogoh, Yoshikazu Inoue

**Affiliations:** ^1^Clinical Research Center, National Hospital Organization Kinki-Chuo Chest Medical Center, Sakai, Osaka, Japan; ^2^Department of Immunology, Kinki Central Hospital of the Mutual Aid Association of Public School Teachers, Itami, Hyogo, Japan; ^3^IVD Development Unit Medical & Biological Laboratories Co. Ltd., JSR Tsukuba Laboratory, Tsukuba, Ibaraki, Japan; ^4^Department of Respiratory Medicine, National Hospital Organization Osaka Toneyama Medical Center, Toyonaka, Osaka, Japan; ^5^Department of Respiratory Medicine and Clinical Immunology, Osaka University Graduate School of Medicine, Suita, Osaka, Japan

## Abstract

**Background:**

We have previously analysed serum autoantibody levels in patients with idiopathic pulmonary fibrosis (IPF), idiopathic nonspecific interstitial pneumonia (iNSIP), and healthy controls and identified the autoantibody against anti-myxovirus resistance protein-1 (MX1) to be a specific autoantibody in iNSIP. We found that a higher anti-MX1 autoantibody level was a significant predictor of a good prognosis in patients with non-IPF idiopathic interstitial pneumonias. In this retrospective study, we sought to clarify the prognostic significance of the anti-MX1 autoantibody in IPF.

**Methods:**

We measured anti-MX1 immunoglobulin (Ig) G, IgA, and IgM autoantibody levels by enzyme-linked immunosorbent assay in serum collected at the time of diagnosis from 71 patients with IPF diagnosed according to the 2018 IPF guideline. The gender-age-physiology (GAP) index was calculated in each case.

**Results:**

The study population (59 men and 12 women) had a median age of 67 years. Serum anti-MX1 IgG and IgA autoantibody levels correlated positively with GAP stage (*p* < 0.05). Univariate Cox proportional hazards regression analysis did not identify an elevated anti-MX1 IgG, IgA, or IgM autoantibody level as a significant prognostic factor; however, a higher anti-MX1 IgA autoantibody level heralded significantly poorer survival after adjustment for GAP stage (*p*=0.030) and for percent forced vital capacity and modified Medical Research Council score (*p*=0.018). Neither the anti-MX1 IgG autoantibody nor the IgM autoantibody could predict survival after these adjustments.

**Conclusions:**

The serum anti-MX1 IgA autoantibody level is a significant prognostic factor in IPF. Further studies are needed to clarify the pathophysiological role of this autoantibody in IPF.

## 1. Introduction

Idiopathic pulmonary fibrosis (IPF) is a progressive fibrotic lung disease of unknown etiology [1–3] with a variable course. IPF usually progresses slowly and insidiously; however, some patients experience rapid deterioration, known as acute exacerbation (AE), which is often fatal [4–6]. Predictors of the prognosis in patients with IPF include gender, age, physiological parameters at the time of diagnosis, and a change in forced vital capacity (FVC) after diagnosis [2]. Physiological parameters also predict the occurrence of AE in IPF [4, 6].

The ATS/ERS/JRS/ALAT guideline for IPF highlights the importance of serum markers for diagnosis, predicting the prognosis and assessing the response to treatment [2]. Krebs von den Lungen (KL)-6 [7] and surfactant apoprotein (SP)-D [8] are well-known serum biomarkers of disease severity, with a higher level of either marker heralding a poor prognosis. However, these markers are not specific for the idiopathic interstitial pneumonias (IIPs) [7, 8] and cannot detect differences in the pathophysiology of these disorders.

We have previously identified anti-myxovirus resistance protein (MX) 1 to be an autoantibody specific for idiopathic nonspecific interstitial pneumonia (NSIP) using protein microarrays [9]. MX proteins belong to a group of GTPases induced by type I interferons (IFNs) that include IFN-*α* and IFN-*β* and are involved in the control of intracellular pathogens [10]. Humans express two types of MX protein, MX1 and MX2, which are encoded by the MX1 and MX2 genes, respectively, on chromosome [9]. MX1 is induced by viral infection and exerts an antiviral action [11] via its GTPase activity. MX1 is also involved in apoptosis [12] and cell motility [13].

We have also found that a positive anti-MX1 autoantibody test for at least one of the three subclasses of antibody (immunoglobulin (Ig) G, IgA, and IgM) in serum [9] predicts a good prognosis in patients with non-IPF IIP after adjustment for modified interstitial lung disease (ILD)-gender-age-physiology (GAP) stage [14, 15]. However, the clinical significance of the serum level of this autoantibody in patients with IPF has not been clarified. Therefore, in this study, we evaluated the relationship between the serum anti-MX1 autoantibody level and disease severity and whether it can predict survival and AE in patients with IPF.

## 2. Methods

### 2.1. Subjects and Diagnosis of IPF

At our institution, bronchoscopy is performed in most patients suspected to have IIP provided that they are able to tolerate pulmonary function tests (PFTs). We retrospectively reviewed the National Hospital Organization Kinki-Chuo Chest Medical Center (KCCMC) database [16] and identified 231 consecutive patients with IIP in whom bronchoalveolar lavage (BAL) with/without transbronchial lung biopsy (TBLB) was performed between 2005 and 2009 [6]. Ninety-four of the 231 cases were diagnosed to have IPF according to the 2011 ATS/ERS/JRS/ALAT guideline for IPF [2]. Serum samples obtained at the time of diagnosis were available for 73 of the 94 cases. After exclusion of two patients who had AE at the initial diagnosis of IPF, 71 cases were enrolled. IPF was reconfirmed in all 71 cases according to the 2018 ATS/ERS/JRS/ALAT guideline [3].

IPF was diagnosed based on high-resolution computed tomography (CT) scans with/without pathological findings in surgical lung biopsy specimens in addition to clinical findings [2]. Most of IPF patients with usual interstitial pneumonia pattern on high-resolution CT scans underwent bronchoscopy similar to those with other patterns in our institute because discrimination between IPF and hypersensitivity pneumonitis is difficult as Ohshimo et al. reported [17].

The retrospective study was approved by the KCCMC Ethics Committee on May 5, 2014 (approval 463). The need for informed consent was waived because all subjects had consented to collection of blood for measurement of markers in serum and to use their clinical information for research purposes at the time of diagnosis of IPF (approval 365).

### 2.2. Clinical Findings at Diagnosis

We retrospectively reviewed the demographic and clinical findings for each patient at the time of diagnosis of IPF, including age, gender, body mass index, smoking status, modified Medical Research Council (mMRC) score [18], PFTs, and serum markers. PFTs, including FVC and diffusing capacity of carbon monoxide (DLco), were performed using a Chestac 8080 spirometer (Chest, Tokyo, Japan). All clinical data for each patient with IIP were collected from the medical records. The severity of IPF at the time of diagnosis was evaluated by GAP stage as defined by Ley et al. [14].

### 2.3. Measurement of Anti-MX1 Autoantibodies in Serum

The serum anti-MX1 autoantibody level was measured in each case by enzyme-linked immunosorbent assay (ELISA) as previously described [9] using previously prepared recombinant human MX1 protein. The antibody subclass (IgG, IgA, or IgM) was measured using the respective anti-human IgG, IgA, or IgM antibody conjugated to peroxidases as the secondary antibody (MBL codes 208, 210, and 212, respectively). Absorbance (optical density; OD) at 450–620 nm was measured and used as the serum autoantibody level for analysis. Serum anti-MX1 autoantibody (IgG, IgA, and IgM) levels were measured in healthy volunteers, and the cutoff values for each antibody subclass (OD) were determined to be 0.237, 0.312, and 0.450, means plus 6 standard deviations, as reported previously [9].

### 2.4. Measurement of Other Serum Markers

Serum KL-6 and SP-D levels were measured using commercial ELISA kits (KL-6, Eisai, Tokyo, Japan; SP-D, Kyowa Medex, Tokyo, Japan). [19] The cutoff levels for KL-6 and SP-D were 500 U/mL and 110 ng/mL, respectively.

### 2.5. Diagnosis of AE in Patients with IPF

AE of IPF was diagnosed according to the Japanese Respiratory Society criteria [20, 21] as follows. (1) The following three conditions should be satisfied within the course of one month in a patient with chronic IPF: progressively worsening dyspnea, new ground-glass opacities evident on high-resolution CT scans superimposed on background reticular or honeycomb patterns, and a reduction in resting PaO_2_ by more than 10 Torr compared with previous measurements. (2) Exclusion of an obvious cause of acutely impaired respiratory function, such as infection, pneumothorax, cancer, pulmonary embolism, or congestive cardiac failure. Apparent infections were excluded by measuring antibodies for *Mycoplasma pneumoniae* and *Chlamydia pneumoniae* in paired sera, *β*-D glucan, and cytomegalovirus antigen tests, and bacterial cultures of blood and sputum. Congestive heart failure was excluded by echocardiography and pulmonary embolism by contrast CT and/or echo Doppler examination.

### 2.6. Statistical Analysis

Data for continuous variables are shown as the median and interquartile range (IQR) and those for categorical variables as the number and percentage. Associations between various markers of disease severity were evaluated by Spearman rank correlation. The clinical significance of each parameter, including the serum anti-MX1 autoantibody level, as a predictor of survival and occurrence of AE at the time of diagnosis of IPF was determined by Cox proportional hazards regression analysis. All statistical analyses were performed using SPSS version 26 for Macintosh (IBM Corp, Armonk, NY, USA). A *p* value <0.05 was considered statistically significant.

## 3. Results

### 3.1. Patient Demographics

The patient demographics are given in Table 1. IPF was diagnosed from surgical lung biopsy specimens in 35 of the 71 cases. The median age at diagnosis was 67 years (IQR, 61–72). Thirty-three patients had GAP stage I, 32 had stage II, and 4 had stage III. The median serum anti-MX1 IgG, IgA, and IgM autoantibody levels (OD) were 0.166 (IQR, 0.129–2.400), 0.124 (0.093–0.165), and 0.063 (0.046–0.091), respectively. The serum anti-MX1 IgG, IgA, and IgM autoantibody levels exceeded the cutoff value in 18 cases (25.4%), 4 (5.6%), and 0 (0%), respectively (Table 1). Prednisolone, immunosuppressants, and pirfenidone were administered before AE in 16 patients, 11 patients, and 11 patients, respectively. Frequency of these treatment was not associated with positivity of serum anti-MX1 IgG and IgA autoantibody levels (Tables 2-3).

### 3.2. Outcome in IPF and Serum Anti-MX1 IgG and IgA Autoantibody Levels

**Figure 1:**
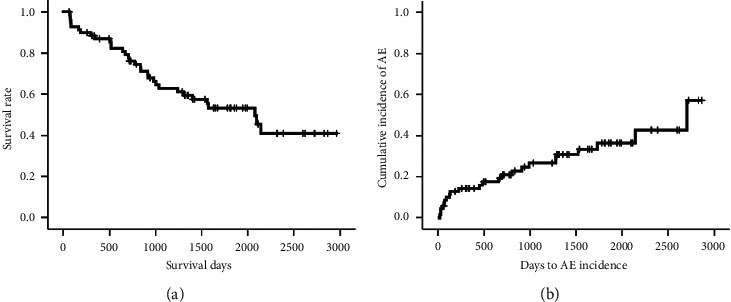
Survival (a) and incidence of acute exacerbation (AE) (b) in patients with idiopathic pulmonary fibrosis (IPF). Median survival time was 2079 days (a) and median interval from diagnosis to AE was 2707 days (b).

**Figure 2:**
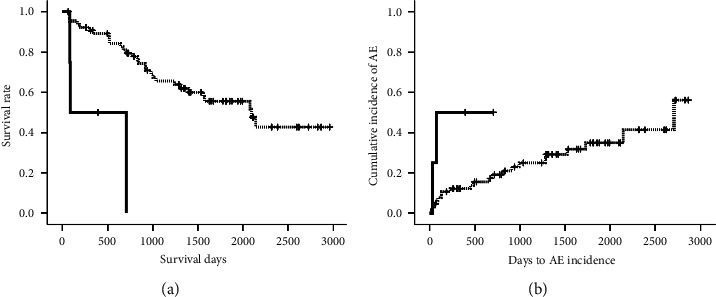
Survival (a) and incidence of acute exacerbation (b) in patients with idiopathic pulmonary fibrosis (IPF). Survival of IPF with higher levels (>0.312; *n* = 4, bold line) of serum anti-MX1 IgA autoantibody was significantly worse than that with lower levels (≤0.312; *n* = 67, dotted line) (log-rank test, *p* < 0.001). AE occurred significantly more frequently in IPF with higher levels of serum anti-MX1 IgA autoantibody (>0.312; *n* = 4, bold line) than in IPF with lower levels (≤0.312; *n* = 67, dotted line) (log-rank test, *p*=0.035).

Median survival time was 2079 days (Figure 1(a)), and median interval from diagnosis to AE was 2707 days (Figure 1(b)). Survival of IPF with higher levels (>0.312) of serum anti-MX1 IgA autoantibody was significantly worse than that with lower levels (≤0.312) (Figure 2(a); log-rank test, *p* < 0.001). AE occurred significantly more frequently in IPF with higher levels of serum anti-MX1 IgA autoantibody (>0.312) than in IPF with lower levels (≤0.312) (Figure 2(b); log-rank test, *p*=0.035). Higher serum levels (>0.237) of anti-MX1 IgG autoantibody were not associated with survival and incidence of AE in IPF patients (log-rank test; *p*=0.159 and *p*=0.368, respectively; data not shown).

### 3.3. Association between Anti-MX1 Autoantibody Level and Other Markers of Disease Severity

Serum anti-MX1 IgG and IgA autoantibody levels were significantly associated with GAP stage. There was a significant correlation of the serum anti-MX1 IgG autoantibody level with %DLco (*p*=0.016) and with the percentage of neutrophils in BAL (Table 4; *p*=0.028). There was also a significant relationship between the serum anti-MX1 IgA autoantibody level and the percentage of lymphocytes in BAL (Table 4; *p*=0.013). High-resolution CT patterns were not associated with serum anti-MX1 IgG and IgA autoantibody levels (Table 4).

### 3.4. Prognostic Factors for IPF Identified in a Cox Proportional Hazards Model

In univariate analysis, a higher mMRC score (≥2), lower %FVC and %DLco values, GAP stage II or III, higher KL-6, and SP-D levels, and a higher percentage of neutrophils in BAL fluid were identified to be significant poor prognostic factors (Table 5). Multivariate analysis with stepwise selection of parameters that had been found to be significant in univariate analysis revealed %FVC (*p*=0.001) and mMRC score (*p*=0.006) to be significant prognostic factors.

### 3.5. Prognostic Significance of the Serum Anti-MX1 Autoantibody Level in IPF

Univariate Cox proportional hazards regression analysis identified an IgA autoantibody level >0.312 to be a significant poor prognostic factor. A higher serum anti-MX1 IgA autoantibody level and an anti-MX1 IgA autoantibody level >0.312 were also found to be significant poor prognostic factors (*p*=0.018 and *p*=0.003, respectively) after adjustment for mMRC and %FVC (Table 6) and for GAP stage (*p*=0.030 and *p*=0.011, respectively; Table 6).

### 3.6. Predictors of AE in Patients with IPF

Predictors of AE were sought in a manner similar to that used for prognostic factors. Multivariate analysis with stepwise selection revealed that a higher %FVC (*p*=0.012) and a higher mMRC score (*p*=0.012) were significant predictors of AE (Table 7).

Serum levels of anti-MX1 IgG, IgA, and IgM autoantibodies were not found to be significant predictors of AE in univariate analysis. However, an anti-MX1 IgA autoantibody level >0.312 was a significant predictor of AE after adjustment for a higher %FVC and a higher mMRC score (*p*=0.028; Table 8), but not after adjustment for GAP stage.

## 4. Discussion

In this study, we found that the anti-MX1 IgA autoantibody level was a significant predictor of a poor prognosis in patients with IPF after adjustment for GAP stage. Multivariate analysis with the stepwise method using various background parameters also identified the anti-MX1 IgA autoantibody to be a significant poor prognostic factor. However, although we found evidence suggesting that the anti-MX1 IgA autoantibody level could predict both survival and occurrence of AE in patients with IPF, we could not clarify the pathophysiological role or mechanism of production of this antibody.

Although the pathophysiology of IPF has not been elucidated, it is thought that dysregulated recovery from lung injury caused by apoptosis of alveolar epithelial cells (AECs) in association with genetic factors and secondary stimulation is a fundamental event [22]. Pulmonary fibrosis develops as a result of aberrant and uncontrolled healing after injury. Upregulation of apoptosis and hyperplasia of AECs is usually observed in the alveolar epithelium of patients with IPF [23]. Hamano et al. found immunohistochemical evidence that MX1 was upregulated in hyperplastic AECs in IPF [9].

MX1 is induced in response to stimulation by type I IFN, and increased MX1 expression in IPF suggests elevation of the type I IFN response. However, in vitro [24, 25] and in vivo [26] studies have demonstrated an association between pulmonary fibrosis and a decreased response to type I IFN. The efficacy of orally administered IFN-*α* for IPF also suggests a reduced type I IFN response in IPF [27], although this has yet to be confirmed. Genetic abnormality is associated with a reduced type I IFN response in IPF. Toll-like receptor (TLR) 3 [28, 29] binds to endogenous mRNA and viral dsRNA, which produces type I IFN and MX1 downstream. Patients with IPF and the TLR3 L412F variant showed accelerated decline in FVC and increased mortality possibly due to reduced production of type I IFN [30]. The engulfment and motility (ELMO) domain containing 2 (ELMOD2) [31] was reported to be a candidate gene for susceptibility to IPF [31]. ELMOD2 is essential for dsRNA-induced activation of the TLR3 pathway and production of MX1 protein [31]. Hence, hypofunctional TLR3 mutations and ELMOD2 gene deficiency [32] in IPF may contribute to progressive lung fibrosis via a decreased type I IFN response, which suggests that induction of MX1 expression in IPF [9] is not mediated by type I IFN.

Ortiz et al. reported that the endoplasmic reticulum (ER) stress response increases MX1 mRNA and induces apoptosis in prostate cancer cells [33]. It is also known that damaged DNA upregulates IFN-stimulated genes [34, 35]. In patients with IPF, hyperplastic AECs show severe ER stress, DNA damage, and consequent apoptosis [22, 36]. Therefore, ER stress and DNA damage possibly enhance MX1 expression and cause apoptosis of AECs in IPF without intervention of type I IFN. It is thought that the anti-MX1 autoantibody is produced against MX1 released from apoptotic AECs [37]. Whether anti-MX1 autoantibodies have a physiological function or not has not been clarified; however, we suspect that an anti-MX1 autoantibody cannot neutralize MX1 due to the intracellular localization of MX1 and that the presence of the anti-MX1 autoantibody in serum reflects the presence and severity of chronic alveolar epithelial injury. The higher the serum anti-MX1 autoantibody level, the more severe the epithelial injury and the worse the prognosis of IPF. The pathophysiology of AE-IPF involves acute progression of chronic epithelial damage [4] and whether or not AE occurring may depend on the degree of chronic epithelial damage. The presence of anti-MX1 IgA autoantibody in a patient with IPF suggests a high likelihood of AE.

Hamano et al. defined anti-MX1 autoantibody-positive cases based on IgG, IgA, or IgM autoantibody positivity [9]. In our study, we examined the significance of the three types of anti-MX1 autoantibodies separately and found that the IgA autoantibody best reflected the prognosis and likelihood of AE in IPF. Transforming growth factor (TGF)-*β* coupled with interleukin (IL)-10 has been reported to induce production of IgA antibodies [38]. In IPF, expression of TGF-*β* and IL-10 in lung biopsy specimens is upregulated [39]. Hence, the anti-MX1 IgA autoantibody could be the predominant autoantibody produced in patients with IPF and local elevation of TGF-*β* and IL-10 concentrations. Thus, IPF with higher levels of anti-MX1 IgA autoantibody might show more local production of TGF-*β* and IL-10, more apoptotic AECs in the lung, and progression of pulmonary fibrosis that is more rapid than would be found with lower levels of this autoantibody. Hence, as shown in our study, higher anti-MX1 IgA autoantibody levels in IPF resulted in a poor outcome. Predominance of the IgA autoantibody over the IgG autoantibody as a predictor of survival is consistent with that of IgA antibody against citrullinated protein antigen in IPF [40].

Hamano et al. reported that patients with non-IPF IIP (including NSIP) who were anti-MX1 autoantibody-positive survived for longer than those who were anti-MX1 autoantibody-negative after adjustment for GAP stage [9]. These inconsistent findings regarding the prognostic significance of the anti-MX1 autoantibody might reflect a pathophysiological difference between IPF and NSIP. Jonigk et al. examined expression of remodelling-related genes in biopsy specimens of IPF, NSIP, organizing pneumonia, and pleuroparenchymal fibroelastosis and clarified the genes discriminating each type of IIP [41]. The antibody against melanoma differentiation-associated gene 5 (MDA5), which is a member of the RIG-I family of proteins, which detect cytoplasmic viral infection and are associated with innate immunity and type I IFN similar to MX1 [42], was found to be positive in serum from patients with clinically amyopathic dermatomyositis (CADM) and rapidly progressive interstitial lung disease (ILD) [43, 44]. Moreover, the enhanced type I IFN activity observed in NSIP [45] and CADM-ILDs [46] suggests that production of both antibodies is associated with viral infection. However, whether or not the antibodies against MX1 and MDA5 have a common pathophysiological role in ILDs requires clarification in the future.

This study has some limitations. First, it had a retrospective, single-center design. Second, it included only four patients with IPF and an anti-MX1 IgA autoantibody level >0.312, so the predictive significance of a higher level has yet to be confirmed. However, the absolute anti-MX1 IgA autoantibody level was a significant prognostic factor.

## 5. Conclusion

We conclude that the serum anti-MX1 IgA autoantibody level is a significant predictor of the prognosis and AE in patients with IPF. Further studies are needed to confirm this finding and the pathophysiological role of anti-MX1 autoantibodies in IPF.

## Figures and Tables

**Table 1:** Patient demographics.

Parameters	Frequency (%) or median (IQR)
Gender, male/female	59/12 (83.1/16.9)
Age, years	67 (61–72)
Smoking, y/n	62/9 (87.3/12.7)
Smoking, CS/EX/NS	15/47/9 (21.1/66.2/12.7)
Packyears	
Current smoker	36 (27.75–50.00)
Ex-smoker	45 (32.25–67.13)
Diagnosis of IPF, Clinical/SLB	36/35 (50.7/49.3)
HRCT pattern^†^, UIP/possible/inconsistent	46/21^‡^/4^‡^
BMI	24.9 (23.3–26.3)
mMRC, <2/2≤	48/23 (67.6/32.4)
FVC, %^*∗*^	76.5 (64.6–90.5)
DLco, %^∗∗^	51.1 (37.2–62.0)
GAP stage, I/II/III^∗∗^	33/32/4 (47.8/46.4/5.8)
KL-6, U/mL^*∗*^	849 (587–1203)
SP-D, ng/mL^∗∗^	178 (110–305)
Neutrophils in BAL, %^*∗*^	2.30 (0.80–6.00)
Lymphocytes in BAL, %^*∗*^	7.30 (3.40–12.4)
Prednisolone started before AE	16 (22.5%)
Prednisolone started after AE	17 (23.9%)
Immunosuppressants started before AE^§^	11 (15.5%)
AZP/CyA/CPA	5/5/1
Immunosuppressants started after AE^§^	9 (12.8%)
AZP/CyA/CPA	5/2/2
Pirfenidone started before AE	11 (15.5%)
Pirfenidone started after AE	0 (0%)
Occurrence of AE, yes/no	23/48 (32.4/67.6)
Observation period^#^, days	1289 (516–1879)
Final outcome, alive/dead	39/32 (54.9/45.1)
Anti-MX1 IgG autoantibody	0.166 (0.129–2.400)
Anti-MX1 IgA autoantibody	0.124 (0.093–0.165)
Anti-MX1 IgM autoantibody	0.063 (0.046–0.091)
Anti-MX1 IgG autoantibody, >0.237	18 (25.4%)
Anti-MX1 IgA autoantibody, >0.312	4 (5.6%)
Anti-MX1 IgM autoantibody, >0.450	0 (0%)

CS, current smoker; ES, ex-smoker; NS, nonsmoker; IPF, idiopathic pulmonary fibrosis; SLB, surgical lung biopsy; HRCT, high-resolution computed tomography; UIP, usual interstitial pneumonia; BMI, body mass index; mMRC, modified Medical Research Council score for shortness of breath; FVC, forced vital capacity; DLco, diffusing capacity of carbon monoxide; KL-6, Krebs von den Lungen-6; SP-D, surfactant protein-D; AE, acute exacerbation; AZP, azathioprine; CyA, cyclosporine A; CPA, cyclophosphamide; MX1, myxovirus resistance protein-1; Ig, immunoglobulin. Number of patients: ^*∗*^ (*n* = 70), ^∗∗^ (*n* = 69), the other parameters (*n* = 71). ^§^All patients treated with immunosuppressants underwent prednisolone therapy. None of the patients were treated with triple therapy using prednisolone, azathioprine, and N-acetylcysteine. ^#^Days from diagnosis of IPF to death or last follow-up. All anti-MX1 IgA autoantibody-positive cases were included in the anti-MX1 IgG autoantibody-positive cases. ^†^Median %FVC of IPF patients with UIP, possible UIP, and inconsistent with UIP patterns on HRCT was 76.1%, 83.1%, and 71.4%, respectively. There was no significant difference among the 3 groups by the Kruskal–Wallis test (*p*=0.765). ^‡^Patients with possible UIP pattern and inconsistent with UIP pattern on HRCT were diagnosed as IPF by histological findings of SLB specimens after the multidisciplinary discussion.

**Table 2:** Correlation between titer of anti-MX1 IgG autoantibody and treatment.

Parameters	Anti-MX1 IgG autoantibody, >0.237	Anti-MX1 IgG autoantibody, ≤0.237	*P* value^*∗*^
Number	18	53	
Before AE			
PSL, yes/no	7/11	9/44	0.099
Immunosuppressants, yes/no	5/13	6/47	0.131
Pirfenidone, yes/no	3/15	8/45	1.000
Started after AE			
PSL, yes/no	5/13	12/41	0.751
Immunosuppressants, yes/no	2/16	7/46	1.000
Pirfenidone, yes/no	0/18	0/53	1.000

MX1, myxovirus resistance protein-1; AE, acute exacerbation; PSL, prednisolone. ^*∗*^Fisher's exact test was performed.

**Table 3:** Correlation between titer of anti-MX1 IgA autoantibody and treatment.

Parameters	Anti-MX1 IgA autoantibody, >0.312	Anti-MX1 IgA autoantibody, ≤0.312	*P* value^*∗*^
Number	4	67	
Before AE			
PSL, yes/no	1/3	15/52	1.000
Immunosuppressants, yes/no	1/3	10/57	0.498
Pirfenidone, yes/no	0/4	11/56	1.000
Started after AE			
PSL, yes/no	2/2	15/52	0.241
Immunosuppressants, yes/no	0/4	9/58	1.000
Pirfenidone, yes/no	0/4	0/67	1.000

MX1, myxovirus resistance protein-1; AE, acute exacerbation; PSL, prednisolone. ^*∗*^Fisher's exact test was performed.

**Table 4:** Correlation between titer of anti-MX1 autoantibodies and severity markers of IPF.

Parameters	Anti-MX1 IgG autoantibody	Anti-MX1 IgA autoantibody	Anti-MX1 IgM autoantibody
Anti-MX1 IgG antibody	1	0.585 (<0.001)	0.230 (0.054)
Anti-MX1 IgA antibody	0.585 (<0.001)	1	0.402 (0.001)
Anti-MX1 IgM antibody	0.230 (0.054)	0.402 (0.001)	1
%FVC	−0.209 (0.082)	−0.077 (0.526)	−0.072 (0.556)
%DLco	−0.290 (0.016)	−0.144 (0.239)	−0.115 (0.346)
KL-6	0.211 (0.079)	0.087 (0.475)	0.138 (0.255)
SP-D	0.183 (0.132)	0.094 (0.440)	0.224 (0.064)
Neutrophils in BAL, %	0.263 (0.028)	0.135 (0.266)	0.051 (0.677)
Lymphocytes in BAL, %	−0.060 (0.619)	−0.295 (0.013)	−0.158 (0.191)
HRCT pattern, UIP/possible/inconsistent	0.017 (0.887)	0.023 (0.857)	0.109 (0.307)
GAP stage	0.319 (0.008)	0.272 (0.024)	0.085 (0.489)
mMRC	0.170 (0.157)	0.120 (0.320)	0.058 (0.631)

MX1, myxovirus resistance protein-1; IPF, idiopathic pulmonary fibrosis; FVC, forced vital capacity; DLco, diffusing capacity of carbon monoxide; KL-6, Krebs von den Lungen-6; SP-D, surfactant protein-D; BAL, bronchoalveolar lavage; HRCT, high-resolution computed tomography; UIP, usual interstitial pneumonia; GAP stage, gender, age, and physiology stage; mMRC, modified Medical Research Council score for shortness of breath; Ig, immunoglobulin. Spearman rank correlation was performed, and Rho (*p* value) was shown in each column.

**Table 5:** Univariate and multivariate Cox proportional hazard regression analyses to evaluate prognostic factors.

Parameters	HR	95% CI	*P* value
Univariate analysis			
Gender, male vs. female	0.988	0.405–2.410	0.979
Age	1.023	0.977–1.072	0.328
Smoking, y/n	0.877	0.337–2.283	0.788
Diagnosis of IPF, clinical vs. SLB	1.474	0.732–2.967	0.277
BMI	0.957	0.855–1.071	0.444
mMRC, 2≤ vs. <2	4.946	2.411–10.021	<0.001
%FVC	0.950^*∗*^	0.930–0.970	<0.001
%DLco	0.961^*∗*^	0.939–0.983	0.001
Neutrophils in BAL, %	1.093	1.020–1.172	0.012
Lymphocytes in BAL, %	0.962	0.913–1.014	0.152
GAP stage, II vs. I	6.799	2.960–15.617	<0.001
GAP stage, III vs. I	11.055	2.113–57.840	0.004
KL-6, x100 U/mL	1.056	1.019–1.095	0.003
SP-D, x10 ng/mL	1.020	1.005–1.036	0.009
HRCT pattern			
Possible UIP vs. UIP	1.006	0.476–2.129	0.987
Inconsistent with UIP vs. UIP	1.259	0.292–5.423	0.758
Multivariate analysis using background parameters except for GAP stage			
mMRC, 2≤ vs. <2	2.923	1.366–6.251	0.006
%FVC	0.956^*∗*^	0.936–0.984	0.001

HR, hazard ratio; CI, confidence interval; IPF, idiopathic pulmonary fibrosis; BMI, body mass index; mMRC, modified Medical Research Council score for shortness of breath; %FVC, percent predicted value of forced vital capacity; %DLco, percent predicted value of diffusing capacity of carbon monoxide; GAP stage, gender, age, and physiology stage; KL-6, Krebs von den Lungen-6; SP-D, surfactant protein; HRCT, high-resolution computed tomography; UIP, usual interstitial pneumonia. Prognostic significance of each parameter was evaluated by univariate Cox proportional hazard regression analysis. Multivariate analysis with the stepwise method was performed using all significant parameters except for GAP stage to clarify prognostic factors specific for our cases other than GAP stage. ^*∗*^HRs of %FVC and %DLco less than 1 suggest patients with higher %FVC and higher %DLco survive longer.

**Table 6:** Prognostic significance of anti-MX1 autoantibodies in IPF adjusted by parameters at the diagnosis of IPF.

Parameters	Univariate analysis			Adjusted by Cox analysis using parameters selected in Table 3			Adjusted by Cox analysis using GAP stage (I, II, III)		
	HR	95% CI	*P* value	HR	95% CI	*P* value	HR	95% CI	*P* value
Anti-MX1 IgG Ab	27.21	0.339–2181	0.140	0.317	0.001–68.94	0.676	0.566	0.004–79.27	0.822
Anti-MX1 IgG Ab >0.237	1.707	0.804–3.620	0.164	0.845	0.364–1.903	0.696	0.952	0.465–2.257	0.952
Anti-MX1 IgA Ab	4.243	0.417–43.19	0.222	498.3	2.915–85200	0.018	306.4	1.744–53826	0.030
Anti-MX1 IgA Ab >0.312	7.250	1.997–26.319	0.003	7.602	2.013–28.70	0.003	5.552	1.488–20.717	0.011
Anti-MX1 IgM Ab	0.466	0.002–107.6	0.783	1.330	0.002–1017	0.933	2.231	0.006–800.5	0.789
Anti-MX1 IgM Ab >0.450	NA			NA			NA		

MX1, myxovirus resistance protein-1; Ab, autoantibody; IPF, idiopathic pulmonary fibrosis; GAP stage, gender, age, and physiology stage; HR, hazard ratio; CI, confidence interval; Ig, immunoglobulin. Prognostic significance of each anti-MX1 autoantibodies, definite titer, or positivity more than cutoff values, was evaluated by univariate Cox proportional hazard regression analysis and HR adjusted by GAP stage was also shown.

**Table 7:** Cox proportional hazard regression analysis to predict acute exacerbation in IPF.

Parameters	HR	95% CI	*P* value
Univariate analysis			
Gender, male vs. female	1.074	0.364–3.166	0.897
Age	1.042	0.983–1.104	0.165
Smoking, y/n	0.783	0.249–2.182	0.583
Diagnosis of IPF, clinical vs. SLB	1.583	0.689–3.633	0.279
BMI	1.011	0.884–1.156	0.870
mMRC, 2≤ vs. <2	4.786	2.024–11.316	<0.001
%FVC	0.955^*∗*^	0.932–0.978	<0.001
%DLco	0.971^*∗*^	0.946–0.996	0.025
Neutrophils in BAL, %	1.086	1.009–1.168	0.028
Lymphocytes in BAL, %	0.997	0.949–1.047	0.901
GAP stage, II vs. I	7.671	2.750–21.399	<0.001
GAP stage, III vs. I	5.928	0.642–57.744	0.117
KL-6, x100 U/mL	1.064	1.024–1.106	0.002
SP-D, x10 ng/mL	1.017	1.001–1.034	0.043
HRCT pattern			
Possible UIP vs. UIP	0.972	0.385–2.453	0.951
Inconsistent with UIP vs. UIP	2.500	0.559–11.179	0.231
Multivariate analysis			
mMRC, 2≤ vs. <2	3.076	1.202–7.870	0.019
%FVC	0.965^*∗*^	0.939–0.992	0.012

HR, hazard ratio; CI, confidence interval; IPF, idiopathic pulmonary fibrosis; BMI, body mass index; mMRC, modified Medical Research Council score for shortness of breath; %FVC, percent predicted value of forced vital capacity; %DLco, percent predicted value of diffusing capacity of carbon monoxide; GAP stage, gender, age, and physiology stage; KL-6, Krebs von den Lungen-6; SP-D, surfactant protein; HRCT, high-resolution computed tomography; UIP, usual interstitial pneumonia. Significance of each parameter to predict occurrence of acute exacerbation in IPF was evaluated by univariate Cox proportional hazard regression analysis. Multivariate analysis with the stepwise method was performed using all significant parameters except for GAP stage, KL-6, and SP-D to clarify predictive factors specific for our cases other than GAP stage. ^*∗*^HRs of %FVC and %DLco less than 1 suggests patients with higher %FVC and higher %DLco experience less occurrence of acute exacerbation.

**Table 8:** Significance of anti-MX1 autoantibody to predict acute exacerbation in IPF adjusted by parameters at the diagnosis of IPF.

Parameters	Univariate analysis			Adjusted by Cox analysis using parameters selected in Table 5			Adjusted by Cox analysis using GAP stage (I, II, III)		
	HR	95% CI	*P* value	HR	95% CI	*P* value	HR	95% CI	*P* value
Anti-MX1 IgG Ab	37.14	0.278–4970	0.148	2.506	0.010–614.2	0.743	1.408	0.005–438.8	0.907
Anti-MX1 IgG Ab >0.237	1.504	0.615–3.679	0.371	0.938	0.366–2.408	0.895	0.790	0.291–2.147	0.664
Anti-MX1 IgA Ab	2.423	0.141–41.59	0.542	236.7	0.602–93185	0.073	25.89	0.040–16680	0.324
Anti-MX1 IgA Ab >0.312	4.432	0.978–20.089	0.054	5.097	1.092–23.791	0.038	4.486	0.993–20.264	0.051
Anti-MX1 IgM Ab	0.659	0.002–280.5	0.893	1.384	0.001–1597	0.928	2.688	0.005–1504	0.759
Anti-MX1 IgM Ab >0.450	NE			NE			NE		

MX1, myxovirus resistance protein-1; Ab, autoantibody; IPF, idiopathic pulmonary fibrosis; GAP stage, gender, age, and physiology stage; HR, hazard ratio; CI, confidence interval; Ig, immunoglobulin; Ab, antibody. Significance of each anti-MX1 autoantibodies, definite titer, or positivity more than cutoff values to predict acute exacerbation was evaluated by univariate Cox proportional hazard regression analysis, and HR adjusted by GAP stage was also shown.

## Data Availability

The data used to support the findings of this study are available from the corresponding author upon reasonable request and are not publicly available due to privacy or ethical restrictions.

## Abbreviations

AE: Acute exacerbation
AECs: Alveolar epithelial cells
BAL: Bronchoalveolar lavage
CT: Computed tomography
DLco: Diffusing capacity of carbon monoxide
ELISA: Enzyme-linked immunosorbent assay
ELMO: Engulfment and motility
IFN: Interferon
Ig: Immunoglobulin
IIPs: Idiopathic interstitial pneumonias
IL: Interleukin
ILD: Interstitial lung disease
IQR: Interquartile range
FVC: Forced vital capacity
GAP: Gender-age-physiology
NSIP: Nonspecific interstitial pneumonia
IPF: Idiopathic pulmonary fibrosis
KCCMC: National Hospital Organization Kinki-Chuo Chest Medical Center
KL: Krebs von den Lungen
MX1: Myxovirus resistance protein-1
mMRC: Modified Medical Research Council
PFTs: Pulmonary function tests
SP-D: Surfactant apoprotein-D
TBLB: Transbronchial lung biopsy
TGF-*β*: Transforming growth factor-beta
TLR: Toll-like receptor.

## Supplementary Materials

Supplementary Materials The authors have completed the STROBE reporting checklist and it can be accessed at the publisher's website.
